# IL-36γ is a pivotal inflammatory player in periodontitis-associated bone loss

**DOI:** 10.1038/s41598-019-55595-9

**Published:** 2019-12-17

**Authors:** Alexandra Cloitre, Boris Halgand, Sophie Sourice, Jocelyne Caillon, Olivier Huck, Isaac Maximiliano Bugueno, Fareeha Batool, Jérôme Guicheux, Valérie Geoffroy, Philippe Lesclous

**Affiliations:** 1 Inserm, UMR 1229, RMeS, Regenerative Medicine and Skeleton, Université de Nantes, ONIRIS, Nantes, France; 2 Université de Nantes, UFR Odontologie, Nantes, France; 3 CHU Nantes, PHU4 OTONN Nantes, France; 4 EA 3826 Thérapeutiques cliniques et expérimentales des infections, Nantes, France; 5 INSERM (French National Institute of Health and Medical Research), UMR 1260, Regenerative Nanomedicine (RNM), FMTS, Strasbourg, France; 6 Université de Strasbourg, Faculté de Chirurgie-dentaire, Strasbourg, France; 7 Hôpitaux Universitaires de Strasbourg, Pôle de médecine et chirurgie bucco-dentaire, Department of Periodontology, Strasbourg, France

**Keywords:** Osteoimmunology, Periodontitis

## Abstract

Periodontitis is a prevalent chronic inflammatory disease due to the host response (IL-1β, IL-6, TNF-α and IL-17A) to oral bacteria such as *Porphyromonas gingivalis*. The newer members of the IL-1 family, IL-36s (IL-36α/IL-36β/IL-36γ/IL-36Ra/IL-38) are known to be involved in host defense against *P. gingivalis* in oral epithelial cells (OECs) and are considered as key inflammatory mediators in chronic diseases. The aim of this study was to investigate the potential role of IL-36s in periodontitis. We showed here that *IL-36γ* mRNA gingival expression is higher in periodontitis patients, whereas *IL-36β* and *IL-36Ra* mRNA expression are lower compared to healthy controls. Interestingly, the elevated *IL-36γ* expression in patients is positively correlated with the *RANKL*/*OPG* ratio, an index of bone resorption. *In vitro, IL-36γ* expression was induced through TLR2 activation in primary OECs infected with *P. gingivalis* but not in gingival fibroblasts, the most widespread cell type in gingival connective tissue. In OECs, recombinant IL-36γ enhanced the expression of inflammatory cytokines (*IL-1β*, *IL-6, TNF-α* and *IL-36γ*), of *TLR2* and importantly, the *RANKL*/*OPG* ratio. These findings suggest that IL-36γ could be a pivotal inflammatory player in periodontitis by perpetuating gingival inflammation and its associated alveolar bone resorption and could be a relevant therapeutic target.

## Introduction

Periodontitis is a chronic multifactorial disease resulting from dysbiotic bacterial biofilms that compromise the integrity of the tooth-supporting tissue^1^. Hallmarks of periodontitis are gingival inflammation and irreversible destruction of the alveolar bone supporting the tooth, which may result in severe tooth loss. Periodontitis also contributes to systemic inflammation and increases the patients risk and morbidity associated with diseases such as diabetes mellitus^2^, rheumatoid arthritis (RA)^3^, atherosclerosis^4^, asthma^5^ and adverse pregnancy outcomes^6^. Controlling the disease should therefore have local and general benefits. But it implies a better understanding of the pathogenic mechanisms that are not fully deciphered.

*Porphyromonas gingivalis* is a Gram-negative anaerobic bacteria considered as a key pathogen in the pathogenesis of periodontitis (periopathogen)^7^. It is strongly associated with diseased sites, has various virulence factors such as lipopolysaccharide (LPS) and is able to induce dysbiosis in an ecologically balanced biofilm. Although the primary etiology of periodontitis is bacterial, the most of periodontal destruction is secondary to the host response to the bacterial challenge^8^. The recognition of pathogen-associated molecular patterns (PAMPs) such as LPS by toll-like receptors (TLRs) expressed by host cells stimulates the production of pro-inflammatory cytokines such as interleukin (IL)-1, IL-6, tumor necrosis factor-α (TNF-α), IL-17A and Receptor Activator of Nuclear Factor κ-B Ligand (RANKL), the most major pro-osteoclastogenic cytokine. These pro-inflammatory cytokines perpetuate local inflammation and subsequent alveolar bone resorption directly or indirectly. RANKL binds to its receptor RANK expressed by bone-resorbing cells, the osteoclasts, or their precursors from the monocyte-macrophage lineage, and enhances their recruitment, differentiation, fusion and activity. Osteoprotegerin (OPG), a soluble decoy receptor, inhibits osteoclastogenesis by competing with RANK for interaction with RANKL. Therefore, the increase in the RANKL/OPG ratio is considered a good indicator of alveolar bone resorption activity notably in alveolar bone loss associated with periodontitis^9,10^.

IL-36 cytokines (IL-36s) are new members of the IL-1 family that may play a key role in the immune response to *P. gingivalis* during periodontitis^11^. IL-36 cytokines include three agonists (IL-36α, IL-36β and IL-36γ) and two antagonists (IL-36Ra and IL-38)^12,13^. All these cytokines bind to IL-36R a widely expressed dimeric receptor. The antagonizing binding of IL-38 to this receptor has been shown only in one study^14^. But, unlike the other cytokines, IL-38 has been reported to bind several other receptors. IL-36 receptor is composed of the subunit IL-36R specific to IL-36 (IL-1Rrp2) and of the co-receptor IL-1R accessory protein (IL-1RAcP). This co-receptor is shared by the agonists of the IL-1 receptor family. IL-36 agonists induce an inflammatory response through the IL-36R and activate NF-κB and MAPK pathways, whereas IL-36 antagonists binding to IL-36R do not recruit its co-receptor and inhibit the IL-36 signaling pathway. IL-36s are mainly expressed by epithelial cells in barrier tissues and are involved in host immunity in both innate and acquired responses. A large body of evidence points to a key role of IL-36s in psoriasis, whereas their involvement in Crohn disease and RA is still currently debated^12,13,15^. Increasing evidence suggests that IL-36s are important regulators of host defense against pathogens in the oral mucosa^11,16–18^. In periodontitis, IL-36β and IL-36γ have been detected in the patient’s gingival crevicular fluid, an inflammatory exudate collected within the gingival crevice^19^. *In vitro*, IL-36γ was strongly overexpressed in oral epithelial cells (OECs) in response to *P. gingivalis*^11^. IL-36γ stimulates OECs in an autocrine manner to induce expression of inflammatory mediators (IL-6, IL-8, CXCL1, CCL20), suggesting the presence of IL-36R on these cells^11^. While it has been suggested that IL-36γ, like other inflammatory cytokines including TNF-α and IL-33, may support osteoclastogenesis by enhancing the RANKL/OPG ratio, its role in the alveolar bone loss associated with periodontitis has not yet been investigated^20,21^.

In this context, we hypothesize that IL-36s and IL-36γ in particular, could play a pivotal role in the pathogenesis of periodontitis. The aims of our work were (i) to show the gingival expression pattern of IL-36s and its role in periodontitis using human gingival samples and primary gingival cells, and (ii) to present evidence that IL-36γ support osteoclastogenesis by enhancing *RANKL*/*OPG* expression ratio in OECs.

## Results

### Analyses of IL-36s expression in patients with periodontitis

The demographic and clinical characteristics of 20 periodontitis and 16 healthy controls are summarized in Supplementary Table S1. Compared to healthy controls, periodontitis patients were older (average age 50.5 ± 2.2 vs 21.1 ± 1.2). This age discrepancy between patients and healthy controls, as often in periodontitis-based studies, is explained by the surgical procedure performed to harvest healthy gingival tissues during the extraction of impacted wisdom teeth, that is most often performed in young adults.

**Figure 1:**
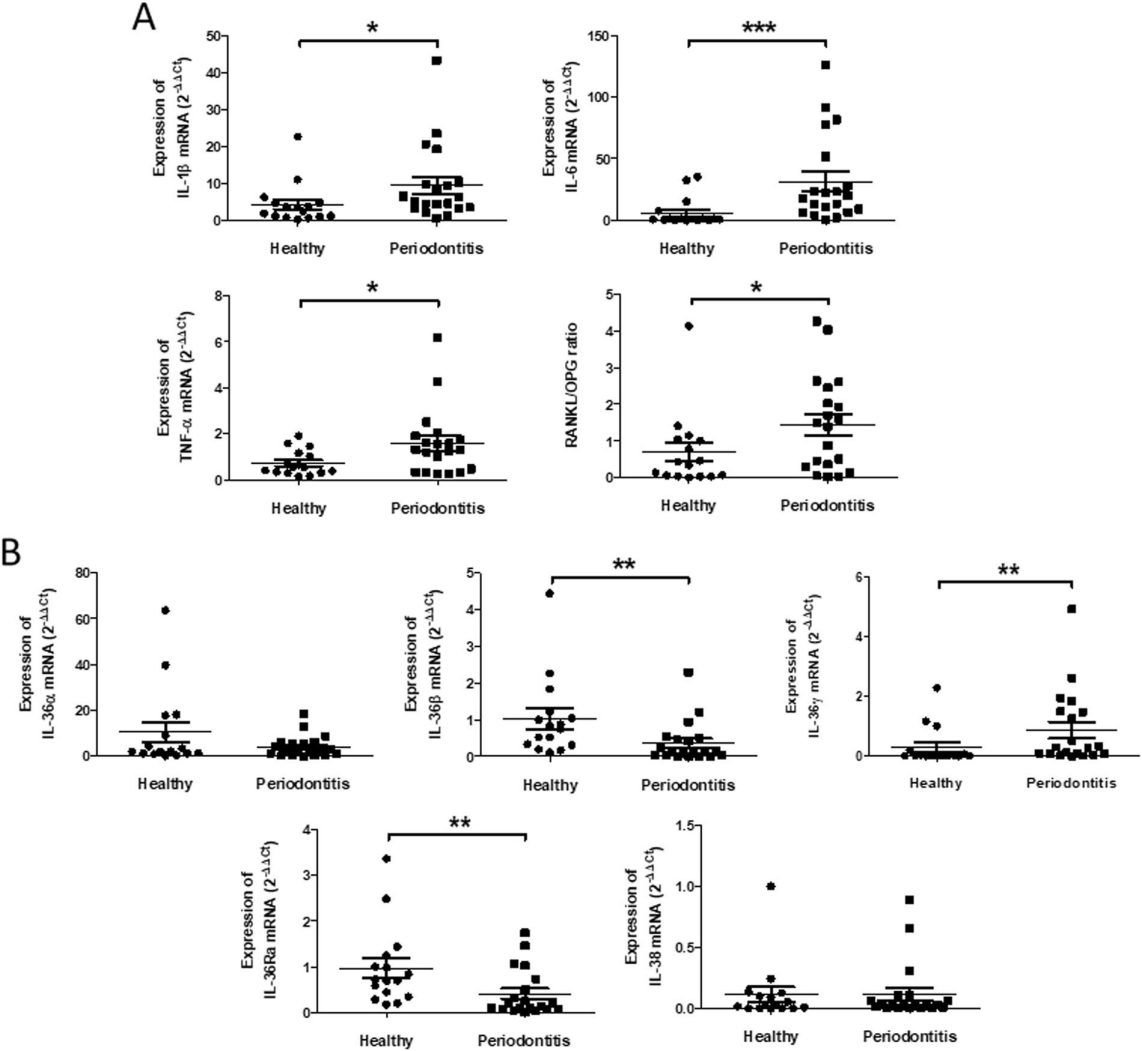
Expression of inflammatory cytokines and *RANKL*/*OPG* ratio in gingival samples of healthy controls and periodontitis patients. (**A**). *IL-1β*, *IL-6*, *TNF-α*, *RANKL* and *OPG* mRNA expression were measured in healthy controls and periodontitis patients by RT-qPCR. *RANKL/OPG* ratio was determined from quantification of *RANKL* and *OPG* expression. (**B**). Expression of the *IL-36* family members was measured by RT-qPCR. Data are shown as mean ± s.e.m; *n* = 16 healthy controls; *n* = 20 periodontitis patients; *p < 0.05; **p < 0.01; ***p < 0.001.

mRNA expression of inflammatory cytokines *IL-1β*, *IL-6*, *TNF-α* and the *RANKL*/*OPG* mRNA ratio were increased in periodontitis patients compared with gingival samples of healthy controls (*p* < 0.05) (Fig. 1), matching the clinical diagnosis of periodontitis (Supplementary Table S1).

Transcript analyses by RT-qPCR revealed that the five IL-36s were expressed in human gingival samples irrespective of their clinical status. Interestingly, *IL-36γ* mRNA expression was found to be the most altered by the clinical condition. A significant increase (3-fold, *p* < 0.01) in *IL-36γ* mRNA was observed in the gingiva of periodontitis as compared to healthy controls, whereas expression of *IL-36β* and *IL-36Ra* was significantly lower (0.4-fold, *p* < 0.01 for both) (Fig. 1). No difference was recorded for *IL-36α* and *IL-38* mRNA expressions.

**Figure 2:**
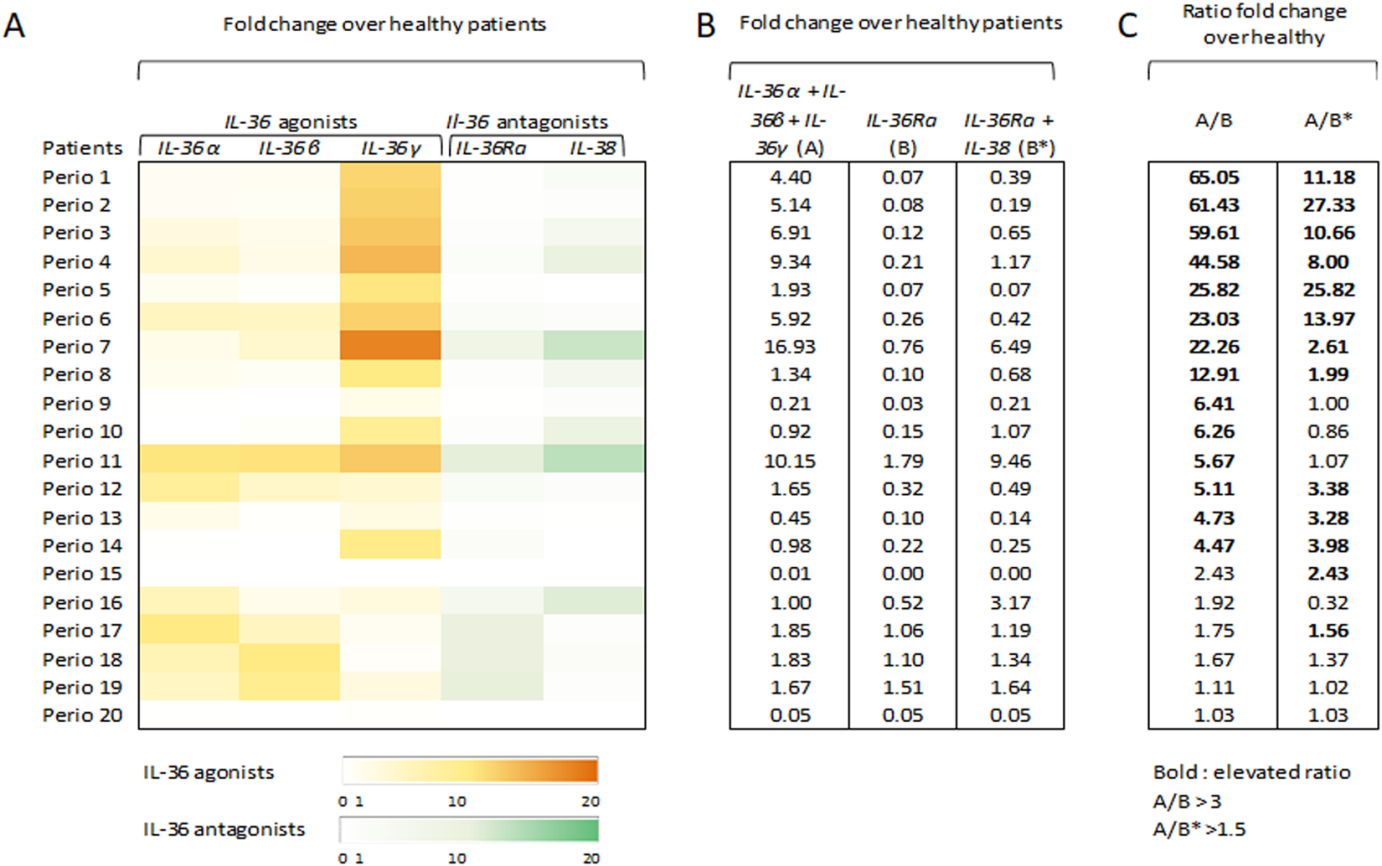
Changes in *IL-36* agonists and antagonists mRNA expression in gingival samples of periodontitis patients. Data are shown in individual patients (*n* = 20; Perio 1 to 20) as fold change over the mean value calculated from the 16 healthy controls. (**A**). *IL-36* agonists (*IL-36α, IL-36β* and *IL-36γ*) are presented in yellow and *IL-36* antagonists (*IL-36Ra* and *IL-38*) in green according to the color scale. (**B**). Sums of *IL-36* agonists (*IL-36α, IL-36β and IL-36γ*) (A), *IL-36Ra* (B) and sums of *IL-36* antagonists (*IL-36Ra* and *IL-38*) (B*) fold changes are presented. (**C)**. *IL-36* agonists/ *IL-36Ra* ratio (A/B) and alternative IL-36 agonists/antagonists (*IL-36Ra* and *IL-38*) ratio (A/B*) are presented. A/B ratio over 3 and A/B* ratio over 1.5 are considered as elevated ratio and are in bold.

To better assess the involvement of the IL-36 signaling in gingival samples from periodontitis patients, we calculated their induction rate (*IL-36* agonists to *IL-36Ra* antagonist). The majority of periodontitis patients (70%) exhibited a ratio over 3, illustrating that expression of *IL-36* agonists is higher than that of antagonists (Fig. 2) and suggesting the activation of the *IL-36* signaling pathway. Then, although the role of IL-38 as IL-36 receptor antagonist is debatable, the alternative ratio of *IL-36* agonists to *IL-36* antagonists, including *IL-36Ra* and *IL-38*, was calculated.in order to compare our results with literature^22^. Most of the patients (78.6%) that exhibit a *IL-36* agonists/ *IL-36Ra* ratio above 3 were also found to show an alternative *IL-36* agonists/ *IL-36* antagonists ratio (*IL-36Ra* and *IL-38*) above 1.5.

We next determined the correlation between the expression of *IL-36β*, *IL-36γ* and *IL-36Ra* in human gingiva and other inflammatory cytokines, receptors and bone resorption markers (Table 1). Of particular interest, in periodontitis patients, *IL-36γ* mRNA is positively correlated with archetypal inflammatory cytokines already known to be involved in periodontitis and inflammatory diseases, i.e., *IL-1β*, *IL-6*, *TNF-α* and *IL-17A* while *IL-36β* or *IL-36Ra* are not correlated with these cytokines. *IL-36γ* mRNA expression in patients was also correlated with those of *TLR2*, *RANKL*, *OPG* and the *RANKL*/*OPG* ratio, unlike *IL-36β* or *IL-36Ra* (*p* > 0.05). Altogether, these results evidenced that *IL-36γ* are correlated with other inflammatory cytokines, receptors and bone resorption markers unlike *IL-36β*/*IL-36Ra* which are not correlated to these factors (Table 1).

**Table 1:** Correlations between expression of *IL-36β*, *IL-36γ*, *IL-36Ra* and cytokines, receptors and bone resorption markers in gingival samples of healthy controls and periodontitis patients.

	*IL-36β*		*IL-36γ*		*IL-36Ra*	
	*p* -value	Spearman r	*p* -value	Spearman r	*p* -value	Spearman r
**Cytokines**						
*IL-1β*	ns, *p *= 0.4067		*, *p* = 0.0217	r = 0.3815	ns, *p *= 0.6783	
*IL-6*	ns, *p *= 0.1873		***, *p* = 0.0001	r = 0.5935	ns, *p* = 0.3478	
*TNF-α*	ns, *p* = 0.5050		*, *p* = 0.0171	r = 0.3951	ns, *p* = 0.8127	
*IL-17A*	**, *p* = 0.0091	r = −0.4609	**, *p* = 0.0035	r = 0.5081	*, *p* = 0.0164	r = −0.4278
**TLRs**						
*TLR2*	ns, *p* = 0.47647		**, *p* = 0.0031	r = 0,5137	ns, *p* = 0,3824	
*TLR4*	ns, *p* = 0.4607		ns, *p* = 0.0604		ns, *p* = 0,2349	
**Bone resorption markers**						
*RANKL*	ns, *p* = 0.0638		***, *p* < 0.0001	r = 0,7431	ns, *p* = 0,0736	
*OPG*	ns, *p* = 0.1679		*, *p* = 0.0459	r = 0,3349	ns, *p* = 0,3692	
*RANKL/OPG*	ns, *p* = 0.3393		**, *p* = 0.0073	r = 0,4397	ns, *p* = 0,2140	

Expression was measured by RT-qPCR. Data are shown as *p*-value and Spearman r coefficient. *n* = 16 healthy controls; *n* = 20 periodontitis patients. ns indicate nonsignificant *p*-values, significant *p*-values are indicated *p < 0.05, **p < 0.01, ***p < 0.001.

**Figure 3:**
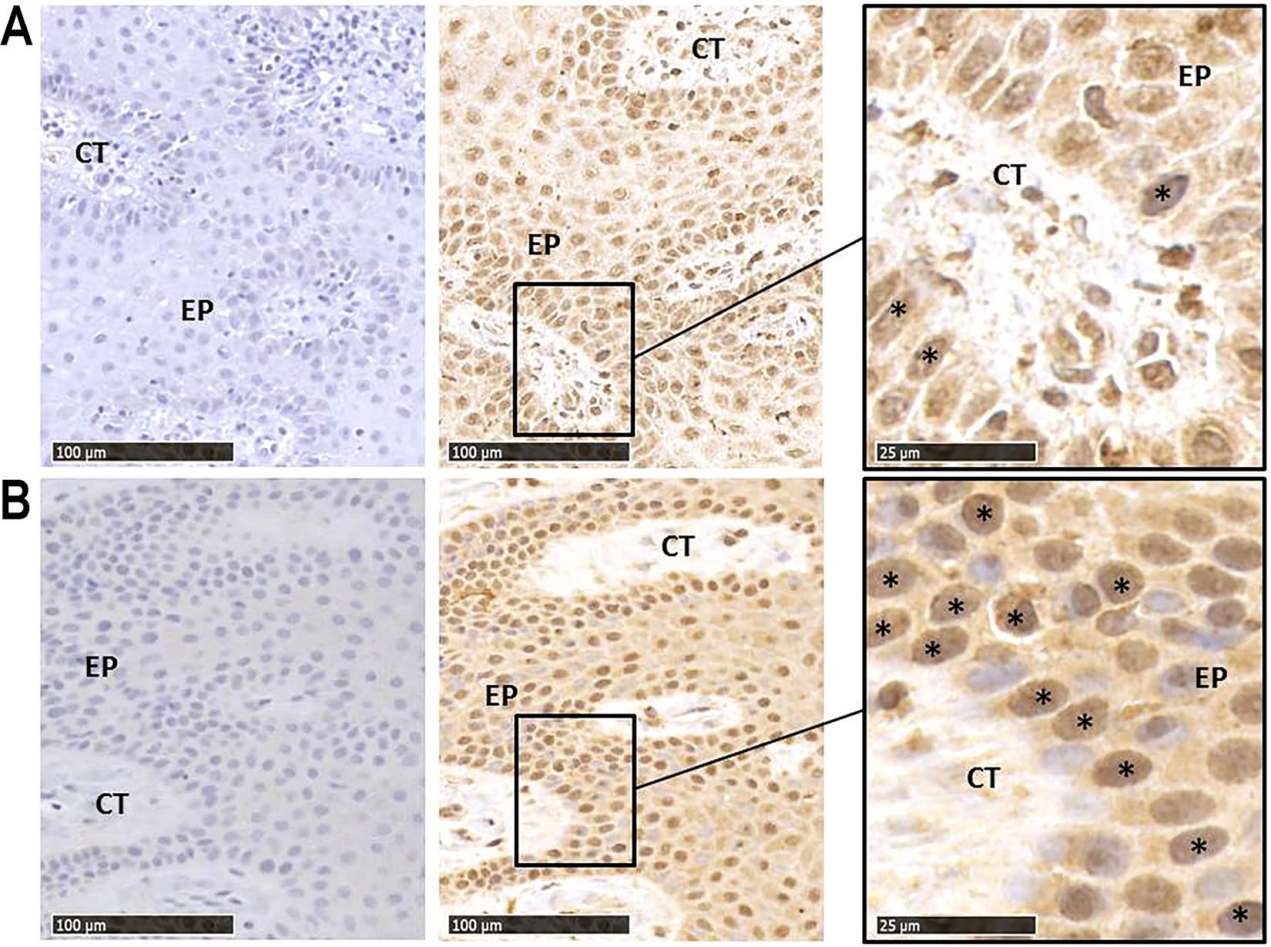
IL-36γ protein expression in gingival samples of healthy controls and periodontitis patients. Serial sections of gingival samples of healthy controls (**A**) and periodontitis patients (**B**) were immunostained with an isotype control antibody or with an anti-IL-36γ antibody. Secondary antibody goat anti-mouse was used. Specific binding was detected using 3,3-diaminobenzidine chromogen. Sections were counterstained with Harris hematoxylin. EP: epithelium; CT: connective tissue, * are positive cells. Scale bar = 100 µm and = 25 µm.

To further confirm mRNA expression data at the level of proteins, we sought to analyze IL-36γ protein expression and to determine its tissue distribution. As expected, immunohistochemistry analyses on gingival samples of periodontitis patients and healthy controls revealed an intense signal in the OECs of the gingival epithelium and a weaker one in the underlying gingival connective tissue mainly composed of GFs (Fig. 3).

These data suggest that variations of IL-36γ expression are representative of the IL-36 signaling activity and that IL-36γ could be worthy of further investigations in the pathogenesis of periodontitis.

### Effect of *P. gingivalis* on IL-36γ expression in human OECs and potential role of TLR2

To further investigate the role of IL-36γ in periodontitis, we investigated the influence of *P. gingivalis* on human established and primary gingival cells (OKF6/TERT2 cell line, primary OECs, and primary GFs) in culture. We performed a time-course of infection over 24 h.

**Figure 4:**
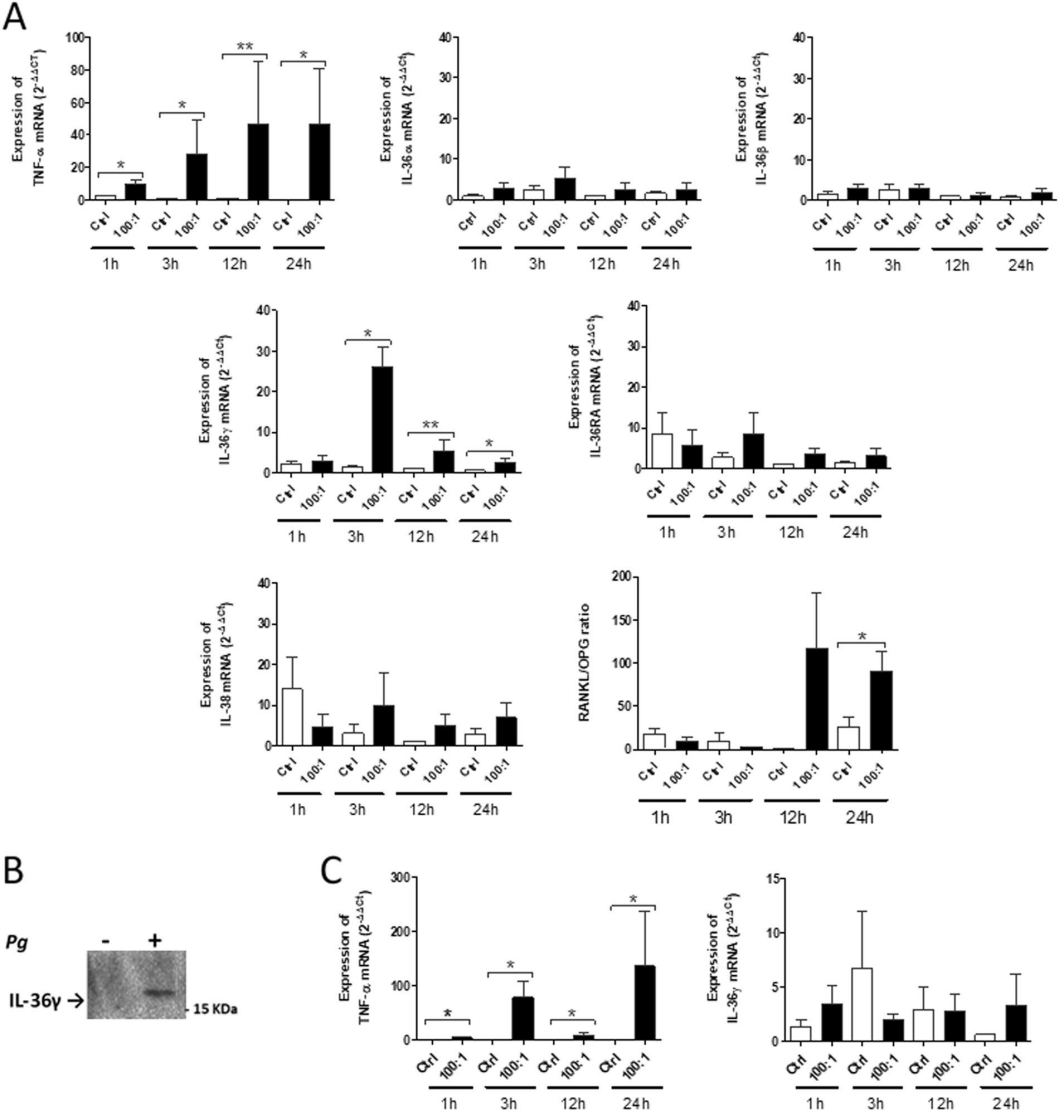
Effect of *Porphyromonas gingivalis* (*Pg*) infection on *IL-36γ* mRNA expression and *RANKL/OPG* expression ratio in human oral epithelial cells (OECs) and in human primary gingival fibroblasts (GFs). (**A**). Human primary OECs were cultured without *Pg* (control; Ctrl) or with *Pg* at 100:1 MOI for 1, 3, 12 or 24 h. *TNF-α* (positive control) and *IL-36γ* mRNA expressions were measured by RT-qPCR and *RANKL/OPG* was determined from quantification of *RANKL* and *OPG* mRNA expression. 4 biological replicates of primary OECs were used. n was used for statistical comparisons; *p < 0.05, **p < 0.01. (**B**). IL-36γ protein expression was assessed by Western blotting in the supernatants of human primary OEC culture. *Pg* – (Ctrl 24 h) and *Pg* + (100:1 MOI 24 h) supernatants were subjected to Western blotting with anti–IL-36γ. The Western blot presented is representative of two independent experiments.(See whole membrane in suplementary Fig. S3) (**C**). Human primary GFs were cultured without *Pg* (control Ctrl) or with *Pg* at 100:1 MOI for 1, 3, 12 or 24 h. *TNF-α* (positive control) and *IL-36γ* mRNA expressions were measured by RT-qPCR. Data are shown as mean ± s.e.m. 3 biological replicates were used for statistical comparisons.

In OKF6/TERT2 cell line, *IL-36γ* mRNA expression was significantly increased by exposure to *P. gingivalis* from 1 h to 24 h at MOI 100:1 (maximum: 17.6-fold at 12 h) (Supplementary Fig. S1). *TNF-α* expression was used as positive control (maximum: 22.0-fold increase at 12 h). The increased expression of *IL-36γ* mRNA after *P. gingivalis* infection was further confirmed in primary OECs from 3 to 24 h at MOI 100:1 (maximum: 19.1-fold at 3 h) (Fig. 4A). TNF-α expression was also used as positive control (maximum: 99.1-fold increase at 24 h). Interestingly, the other IL-36 cytokines were not significantly affected by *P. gingivalis* infection. Of particular relevance to bone loss associated with periodontitis, *P. gingivalis* infection at MOI 100:1 also induced an increase in the *RANKL*/*OPG* ratio (3.5-fold) in primary human OECs, which occurred later than the increase in *IL-36γ* expression (3 h vs 24 h). This pattern of expression strongly suggests that *IL-36γ* is upstream in the signaling cascade and suggests that *IL-36γ* could participate in the increase in the *RANKL*/*OPG* expression ratio in human OECs infected by *P. gingivalis*. Finally, our Western blotting data confirm the presence of IL-36γ protein in the culture supernatant of primary OECs infected by *P. gingivalis* at MOI 100:1 for 24 h but not in that of cells not exposed to *P. gingivalis* (Fig. 4B). IL-36γ protein expression in the supernatant was only investigated at 24 h because it was not detectable before that time.

Because *P. gingivalis* is known to invade deep connective tissue^23^, we finally sought to determine whether *P. gingivalis* infection could also affect the expression level of *IL-36γ* in human primary GFs. According to the lack of IL-36γ immunostaining in the connective tissue of gingival samples (Fig. 3), we found that *IL-36γ* expression was lower in GFs as compared to OECs (average CT of *IL-36γ* mRNA, 33.7 in healthy control GFs vs 25.9 in healthy control OECs). In addition, our data revealed that *P. gingivalis* failed to significantly affect the expression level of *IL-36γ* in human GFs at MOI:100 (Fig. 4C).

**Figure 5:**
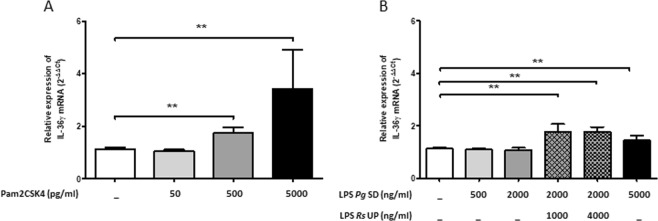
*IL-36γ* mRNA expression in human oral epithelial cells (OECs) stimulated with TLR2 or TLR2 TLR4 agonists with or without TLR4 antagonist for 24 h. Human primary OECs were cultured for 24 h with or without TLR2 agonist (Pam2CSK4) (**A**) and with or without TLR2/TLR4 agonist (LPS-Pg SD) and with or without pretreatment with TLR4 antagonist (LPS-Rs UP) 30 min before stimulation (**B**) at the concentrations indicated. IL-36γ mRNA expression was measured by RT-qPCR. Data are shown as mean ± s.e.m. 5 biological replicates were used for statistical comparisons; **p < 0.01 vs CTL (white bar).

Finally, to further decipher the mechanisms underlying the effects of *P. gingivalis* on *IL-36γ* expression levels, human primary OECs were stimulated with TLR agonizing and antagonizing ligands. After having confirmed that *TLR2* and *TLR4* mRNA were expressed in human primary OECs, cells were stimulated with TLR2 (Pam2CSK4, Fig. 5A) agonists. TLR2 agonist induced a significant increase in *IL-36γ* mRNA. In addition, when using a dual agonist of TLR2/TLR4 in the presence of a specific antagonist of TLR4, we also reported a significant increase in *IL-36γ* mRNA (Fig. 5B). Taken together, these data support the hypothesis that *P. gingivalis* increased *IL-36γ* expression at least through TLR2 activation in human OECs.

### Effects of IL-36γ on the expression levels of inflammatory cytokines, *RANKL/OPG* ratio and *TLR2* in human OECs

**Figure 6:**
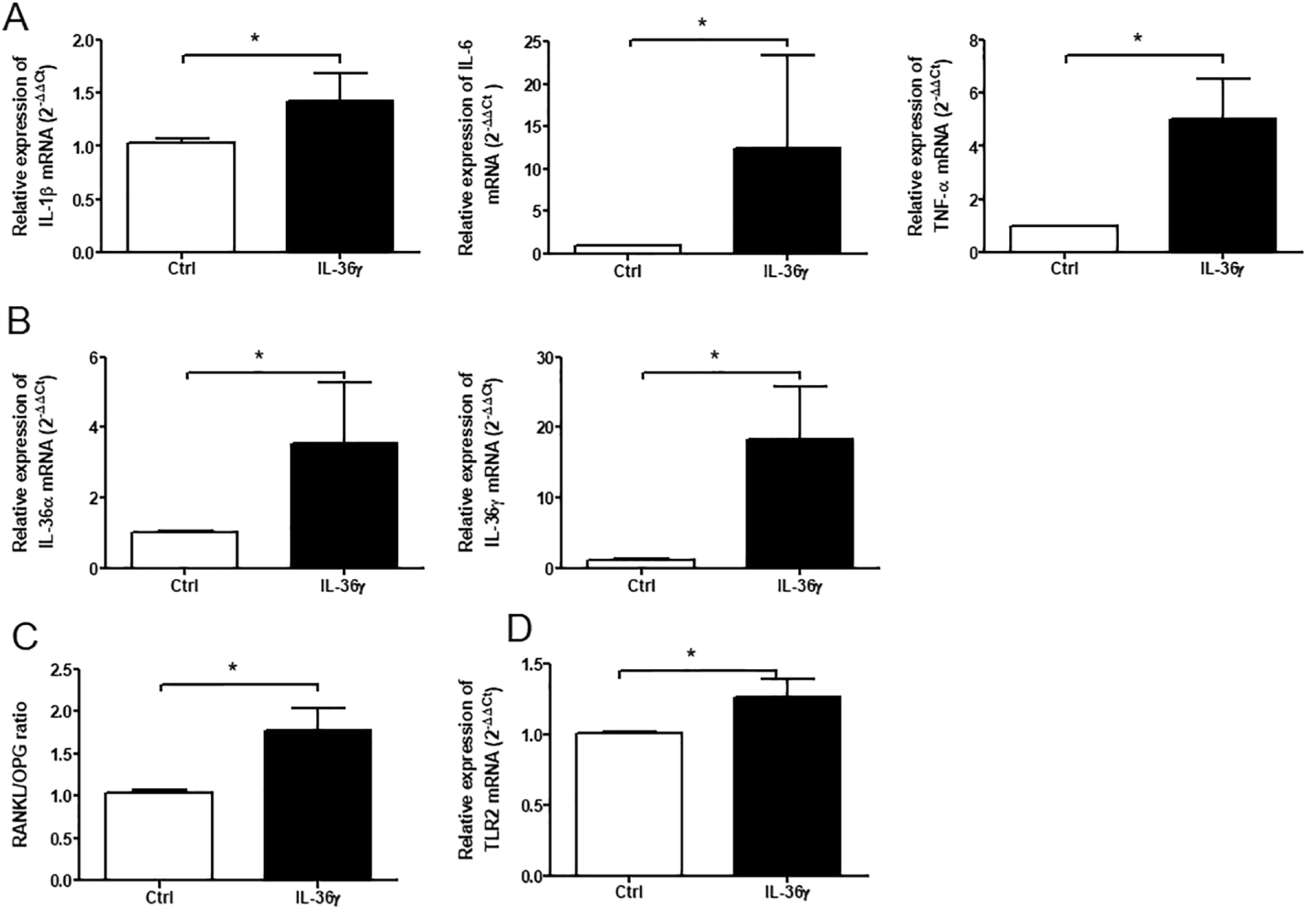
*IL-36γ* increased expression of inflammatory cytokines (*IL-1β, IL-6, TNF-α, IL-36α, IL-36γ*), *RANKL/OPG* ratio and TLR2 in primary human oral epithelial cells (OECs). Human primary OECs were cultured with or without 100 ng/ml of recombinant human IL-36γ for 24 h*. IL-1β, IL-6, TNF-α, IL-36α, IL-36γ, RANKL, OPG, TLR2* mRNA expression were measured by RT-qPCR. The *RANKL/OPG* ratio was determined from quantification of *RANKL and OPG* expression. Data are shown as mean ± s.e.m. 3 biological replicates were used for statistical comparisons; *p < 0.05.

To further elucidate the role of IL-36γ, we were interested in determining the effects of recombinant human IL-36γ (100 ng/ml; 24 h of treatment) on human primary OECs. The RT-qPCR analyses first demonstrated that IL-36γ acted on OECs by enhancing the expression of inflammatory cytokines, which have been clearly established as participating in the pathogenic mechanisms of periodontitis including *IL-1β*, *IL-6* and *TNF-α* with a 1.4-, 12.3- and 5.03-fold increase (Fig. 6A), respectively. In addition, we found that treatment with *IL-36γ* strongly enhanced the expression of *IL-36α* (3.5-fold increase; *p* < 0.05) and *IL-36γ* itself with the highest fold change among all of inflammatory cytokines analyzed in this study (18.4-fold increase; *p* < 0.05) (Fig. 6B). In addition, IL-36γ failed to alter the expression levels of *IL-36β* and those of all *IL-36* antagonists as well (data not shown). Interestingly, and as previously described for *P. gingivalis*, IL-36γ significantly increased the *RANKL*/*OPG* ratio in OECs (3.5-fold increase, *p* < 0.05) (Fig. 6C) as well as *TLR2* (*p* < 0.05, 1.3-fold increase) (Fig. 6D).

## Discussion

In this study, we showed that a majority of periodontitis patients (70%) exhibited an elevated *IL-36* agonists/ *IL-36Ra* antagonist mRNA ratio, suggesting the involvement of IL-36s cytokines in the pathogenesis of the disease. *IL-36γ* was the most highly expressed in the gingiva of patients and its expression was the most affected by periodontitis. Its expression was also increased in human primary OECs upon bacterial challenge with the key periopathogen *P. gingivalis* potentially through TLR2. IL-36γ could perpetuate gingival inflammation by increasing pivotal inflammatory cytokines in periodontitis (*IL-1β*, *IL-6* and *TNF-α*) and alveolar bone resorption through an increase of the *RANKL*/*OPG* ratio in OECs.

In the gingiva, all the *IL-36s* are expressed but only three of them have a modulated expression in periodontitis. Previously, only IL-36β and IL-36γ expressions have been reported *in vivo* in gingival crevicular fluid with a higher IL-36β level in aggressive compared to chronic periodontitis patients^19^. We found that a majority of periodontitis patients (70%) had a high *IL-36* agonists/*IL-36Ra* mRNA ratio. Although the role of IL-38 as IL-36 receptor antagonist is debatable, the alternative ratio of *IL-36* agonists to *IL-36* antagonists, including *IL-36Ra* and *IL-38*, give a similar result. This alternative ratio permit us to compare the tissue expression of *IL-36s* in periodontitis with that of three other chronic inflammatory diseases reported in the literature^22^ (Supplementary Table S2). Sixty-five percent of periodontitis patients exhibited an alternative *IL-36* agonists/antagonists ratio (*IL-36Ra* and *IL-38)* over 1.5. This is higher than in RA or Crohn’s disease (29% and 25%, respectively) in which the role of IL-36s is currently debated, and smaller than in psoriasis (93%) in which IL-36s are key cytokines. This considerably strengthens the hypothesis of a promising role for IL-36s in periodontitis. The increased expression of *IL-36γ* mRNA is the only common hallmark between these diseases. Within periodontitis, an inter-individual variability was observed. This probably limits the use of *IL-36γ* as a diagnostic biomarker, but this strongly suggests the existence of subgroups within periodontitis patients, which lays foundations for personalized medicine. This also raised the question of whether host modulation therapy blocking IL-36s could be relevant in the management of the disease in the subgroup of patients with an elevated *IL-36* agonists/*IL-36Ra* ratio.

OECs are the main gingival cells that express IL-36γ in periodontitis. These cells not only act as a physical barrier against periopathogen invasion, but are also immune contributors in initiating the innate immune defense of the host. In particular, they produce pivotal pro-inflammatory cytokines in periodontitis such as IL-1β, IL-6 and TNF-α in response to bacterial challenge with the key periopathogen *P. gingivalis* through the NFκB and MAPK signaling pathways^24,25^. In accordance with Huynh *et al*.^11^, we reported that OEC infected by these bacteria strongly overexpressed IL-36γ not only in an established OEC line (OKF6 cells), but also in primary OECs isolated from human gingiva. Interestingly, this overexpression occurred early in the time course of infection, raising the hypothesis of a role for IL-36γ in the initiation of the host response to *P. gingivalis*. This hypothesis is in line with a recent report describing IL-36γ as an alarmin for surrounding cells with respect to its release by dying cells and its pro-inflammatory properties^26^. Unlike *IL-36γ*, the expression of other *IL-36 isoforms* by OECs remains unaffected by *P. gingivalis* infection, as previously shown^11^. The distinct variations of IL-36α, IL-36β and IL-36γ expression in inflammatory diseases are documented but not fully understood. Although these three isoforms are processed differentially by neutrophils and skin resident cell proteases^27,28^, they act through the same receptor and seem to have redundant biological effects^12,29^.

The other main gingival resident cells are GFs, which also participate in the immune response to oral bacteria by producing pro-inflammatory cytokines including IL-6 and IL-8^30^. In this study, we reported that *IL-36s* mRNA expression was not altered in human primary GFs infected with *P. gingivalis*. This is in accordance with our immunohistochemical findings, which indicated that GFs were not the main producers of *IL-36s* in the periodontitis gingiva. This is also in agreement with Jang *et al*., who reported that in contrary to less pathogenic bacteria, *P. gingivalis* induced marginally or suppressed the inflammatory cytokine response in GFs, resulting in the persistence of bacteria within periodontal tissue that perpetuates the chronicity of periodontitis^23^.

IL-36s expression has been shown to be induced by TLR signaling in several cell types including OECs^11,12^. *P. gingivalis* LPS can activate host cells to induce pro-inflammatory cytokines through TLR2 and/or TLR4^31–33^. In human gingival samples we showed that *IL-36γ* mRNA expression was positively correlated with *TLR2* but not with *TLR4* and that LPS *P. gingivalis* stimulates *IL-36γ* expression in primary OECs potentially via TLR2. Our results are in line with those of Huynh *et al*., who reported that the stimulation of OECs with the TLR2 synthetic agonist FSL-1 resulted in an induction of IL-36γ expression upon the regulation of IRF6 and IRAK1^11^. We also showed that IL-36γ increases the expression of *TLR2* and the *RANKL*/*OPG* expression ratio in OECs. Interestingly, TLR2 is required for *P. gingivalis*-induced inflammatory bone loss in experimental periodontitis in mice^34–36^. Bone resorption associated with periodontitis is RANKL-dependent, as demonstrated *in vivo* by the anti-resorptive effects of a local anti-RANKL antibody administration^36^. Osteoblasts and macrophages have also been shown to be key TLR2-expressing cells driving alveolar bone resorption induced by *P. gingivalis*^35,37^. Activation of TLR2 in osteoblasts by *P. gingivalis* LPS increased RANKL production^37^. Adoptive transfer of TLR2-expressing macrophages into TLR2-deficient mice restored the ability of *P. gingivalis* to induce inflammatory bone *in vivo*, which is TNF-dependent^35^.

Pivotal inflammatory cytokines in periodontitis, namely IL-1β, IL-6, TNF-α and IL-17A, mediate both periodontal inflammation and alveolar bone resorption^8^. We reported that gingival expression of *IL-36γ* in periodontitis patients was positively correlated with expression of these cytokines, suggesting its involvement in the pathogenesis of the disease. We also showed that IL-36γ could perpetuate inflammation in human primary OECs by increasing gene expression of inflammatory cytokines (*IL-1β*, *IL-6*, *TNF-α*, *IL-36α* and *IL-36γ* by self-amplification) and of *TLR2*. This is compatible with a starting action of IL-36γ in periodontitis and could lead to an uncontrolled inflammation cascade by increasing TLR2-induced cytokines. The effects of IL-36γ previously reported in the OEC line are the increase in the production of inflammatory cytokines (IL-6^11^, IL-23p19/EBI3 (IL-39)^17^). It also stimulates the expression of neutrophil (IL-8, CXCL1) and Th17 cell chemokines (CCL20)^11^ and PGLYRP2 antimicrobial proteins^16^. In addition IL-36γ is known to stimulate human dendritic cells and to a lesser extent macrophages to produce chemokines (IL-8, CXCL1, CCL20)^11^. It is noteworthy that, since IL-17A enhances the expression of IL-36γ in human OECs, a strong inflammatory axis between IL-17A and IL-36γ has been suggested in the oral mucosa^11^. The *in vivo* positive correlation evidenced in this study between *IL-36γ* and *IL-17A* mRNA expression corroborates this hypothesis. We have further reported that IL-36γ could perpetuate the alveolar bone resorption related to periodontitis through an increase in the *RANKL*/*OPG* ratio. In periodontitis, gingival expression of IL-36γ was positively correlated with the *RANKL*/*OPG* ratio. In primary OECs, *IL-36γ* was induced prior to the *RANKL*/*OPG* increase during *P. gingivalis* infection, suggesting that IL-36γ could act as an inducer. This was confirmed by stimulating these cells with IL-36γ. OECs produce a basal level of RANKL able to induce osteoclast formation in a co-culture assays with osteoclast precursor cells^38^. IL-36γ-dependent increase of the *RANKL/OPG* expression ratio in OECs could therefore contribute to increase osteoclast differentiation. Further analyses in a co-culture system (OECs/pre-osteoclasts) will be performed to decipher the effect of IL-36γ produced by OECs on the osteoclastogenesis. Regarding the direct effect of IL-36s on osteoclastogenesis, only IL-36α has been studied. Since IL-36 receptor is not present on mature osteoclasts, IL-36 isoforms have no direct effect on these mature cells^39^.

**Figure 7:**
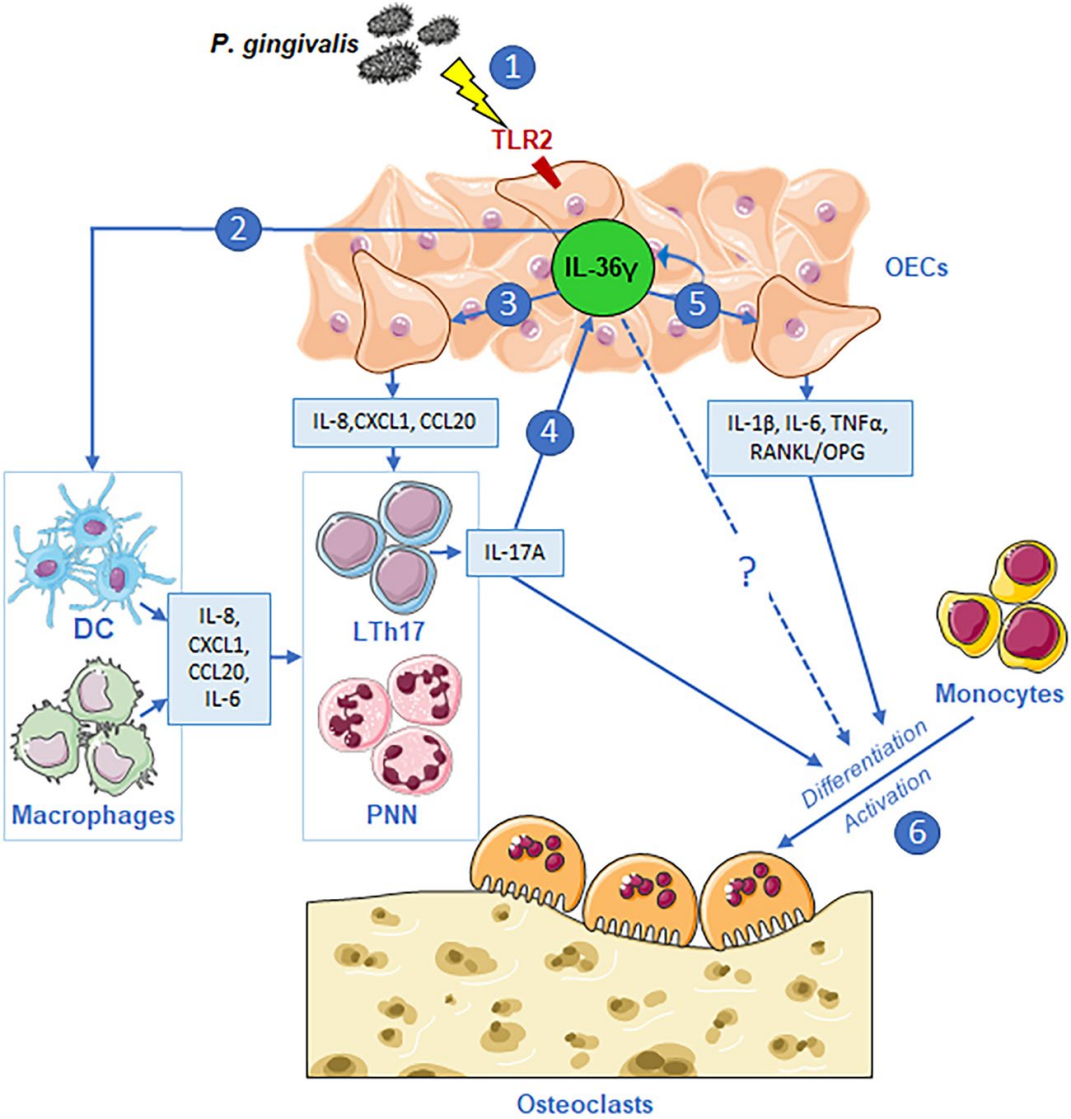
Involvement of IL-36γ in periodontitis pathogenesis. (**1**) *P. gingivalis* induces the expression of IL-36γ by OECs via TLR2 signaling. (**2**). IL-36γ acts on dendritic cells and macrophages to express chemokines attractant for neutrophils (IL-8, CXCL1) and LTh17 lymphocytes (CCL20) and IL-6. (**3**) IL-36γ also acts on OECs by inducing the expression of the same chemokines (IL-8, CXCL1, CCL20). (**4)** Th17 lymphocytes recruited on the inflammation site produce IL-17A, which increases IL-36γ expression by OECs, suggesting an inflammatory axis between these two cytokines. (**5**) IL-36γ also acts on OECs to induce its own expression and expression of IL-136γ, IL-6 and TNF-α, and to increase the RANKL/OPG ratio. (**6**) These cytokines promote the differentiation and activation of osteoclasts that perpetuate alveolar bone resorption. This figure was created using Servier Medical Art templates, which are licensed under a Creative Commons Attribution 3.0 Unported License; https://smart.servier.com.

The mechanisms of IL-36s in periodontitis could be multiple, as highlighted in Fig. 7. Additional studies are needed to better understand the role of IL-36 in periodontitis, and in particular experimental periodontitis in *IL-36R*- or *IL-36γ*-deficient mice could be useful in sustaining its importance in inflammatory bone loss^40^. Moreover, experimental periodontitis could allow the assessment of targeted therapies.

Overall, these findings from human gingival samples and primary gingival cells suggest a pathological involvement of IL-36s, IL-36γ in particular, in the pathogenesis of periodontitis. IL-36γ seems to play a pivotal role in the innate immune response to bacterial challenge with the key periopathogen *P. gingivalis*. IL-36γ is rapidly induced in OECs acting upstream of other cytokines considered as key mediators in periodontitis. Deciphering the mechanisms involving IL-36s in periodontitis is a prerequisite to the development of host modulation therapy blocking IL-36R signaling. The use of recombinant IL-36Ra or receptor-blocking monoclonal antibodies could be promising in periodontitis, as suggested for other inflammatory diseases^41^.

## Materials and Methods

### Gingival sample collection

All enrolled patients provided their written informed consent for study participation. The study was approved by the Institutional Medical Ethics Committee of the Nantes University Hospital (SVTO:DC-2011-1399) and was conducted in accordance with the code of ethics of the World Medical Association (Declaration of Helsinki). The following characteristics were recorded: demography (gender, age), medical history (diseases, medications, tobacco use) and periodontal status (probing pocket depth (PPD), clinical attachment loss (CAL) and bleeding on probing (BOP)). According to the recent periodontal epidemiology working group, periodontitis was defined as PPD ≥ 4 mm, CAL ≥ 3 mm and presence of BOP^42^. Gingival samples were harvested just after an extraction when the mucosa was in excess. Patients were not included in the study if they suffered from systemic diseases that could affect periodontal health (such as diabetes mellitus, immunological disorders, human immunodeficiency virus infections, osteoporosis), or pregnant females and patients who were taking antibiotics, anti-inflammatory or immunosuppressive therapies in the 3 months prior to the dental extractions. The diagnosis of periodontitis was confirmed by RT-qPCR analyses of transcripts coding for inflammatory cytokines such as *IL-1β*, *IL-6*, *TNF-α* and the bone resorption markers *RANKL* and *OPG* (Supplementary Table S3 for primer sequences). The gingival samples were used for RT-qPCR analyses, histology and/or primary cell cultures.

### Oral cell culture

Cell line**:** human OKF6/TERT2 OECs (BWH Cell Culture and Microscopy Core, USA) were amplified in defined keratinocyte-SFM basal medium (K-SFM) with growth supplements (Thermo Fischer Scientific, USA).

Primary cells: human OECs and gingival fibroblasts (GFs) were isolated from gingival samples of healthy controls using the direct explant technique as previously described for the skin^43^. Briefly, cells were characterized by their respective morphology and by RT-qPCR amplification of markers. OECs were tetrahedral and positive for keratin 14 (*KRT14*), whereas GFs were fusiform and positive for *CD90*. OECs were cultured in a serum-free keratinocyte growth medium CnT-07 (CELLnTEC, Switzerland) with 0.1% penicillin-streptomycin. GFs were cultured in Dulbecco’s Modified Eagle’s Medium (DMEM) glutaMAX® (Thermo Fischer Scientific, USA) supplemented with 10% FCS, 1% penicillin-streptomycin. OECs were used at passage 0 for all experiments except for challenging with TLR ligands (passage 1). GFs were used at passage 3.

### Bacterial and strain culture

*P. gingivalis* (ATCC 33277) was cultured at 37 °C on Shaedler agar plated with sheep blood (Becton Dickinson, Germany) in an oxygen-free atmosphere. After 10 days in culture on the day of infection, *P. gingivalis* colonies were selected and resuspended in PBS for cell infection.

### Challenging oral cells with viable *P. gingivalis*

Twenty-four hours before infection with viable *P. gingivalis*, oral cells were washed twice with HBSS and antibiotic-free media was added. The media for OECs was serum-free and the media for GFs was with heat-inactivated serum (30 min at 56 °C). The day of infection, cells were challenged with *P. gingivalis* at the multiplicity of infection (MOI) 10:1 or MOI 100:1.

### Challenging oral cells with TLR ligands or recombinant IL-36γ

OECs were stimulated in a CnT-07 serum-free media with 0.1% penicillin-streptomycin for 24 or 48 h with:

- Pam2CSK4, a synthetic TLR2 agonist (tlrl-pm2s-1, InvivoGen, USA) at 0 to 5000 pg/ml,
- ultrapure LPS from *P. gingivalis* (LPS-*Pg* UP), a TLR4 agonist (tlrl-ppglps, InvivoGen, USA) at 0 to 5000 ng/ml,
- standard LPS from *P. gingivalis* LPS (LPS-*Pg* STD), TLR2 and TLR4 agonists (tlrl-pglps, InvivoGen, USA) at 0 to 5000 ng/ml,
- ultrapure LPS from *Rhodobacter sphaeroides* (LPS-RS), a strong TLR4 antagonist (tlrl-prslps, InvivoGen, USA) at 0 to 10,000 ng/ml with 30 min pretreatment before TLR stimulation,

Primary OECs were stimulated with 100 ng/mL of recombinant human IL-36γ (6835-IL, R&D, USA) in a CnT-07 serum-free media with 0.1% penicillin-streptomycin for 24 h.

### RNA extraction and RT-qPCR analyses

Human gingival tissues were homogenized with the FastPrep® system (MP Biomedicals, USA). Total RNA was isolated from homogenized tissues or cells in culture using the Nucleospin® RNA II kit (Macherey-Nagel, Germany) according to the manufacturer’s instructions. RNA quality and concentration were determined using a NanoDrop® 1000 spectrophotometer (Thermo Fisher Scientific, USA). RNA was reverse-transcribed using SuperScript®III (Thermo Fisher Scientific, USA) according to the manufacturer’s instructions. Relative quantification of gene expression was performed on a CFX96 thermal cycler (BioRad, USA) using the SYBR®Select Master Mix (Applied Biosystems, USA). Primer sequences are indexed in Supplementary Table S3 and were synthesized by Eurofins Scientific® (Luxembourg). For the analyses, *SDHA*, *beta-actin*, and *B2M* were used as endogenous control and the relative gene expression levels were calculated with the 2^−∆∆Ct^ method.

The *IL-36* agonists/*IL-36Ra* ratio was calculated as follows: (*IL-36α* fold increase (mRNA expression in periodontitis vs healthy controls) + *IL-36β* fold increase + *IL-36γ* fold increase) / (*IL-36Ra* fold increase). A patient’s ratio higher than 3 (three agonists/one antagonist) was considered elevated compared to samples of healthy controls. In order to compare with literature, an alternative ratio including *IL-36Ra* and *IL-38* i.e. (*IL-36α* fold increase (mRNA expression in periodontitis vs healthy controls) + *IL-36β* fold increase + *IL-36γ* fold increase) / (*IL-36Ra* fold increase + *IL-38* fold increase), was also calculated as previously described^22^. A patient’s alternative ratio higher than 1.5 (three agonists/two antagonists) was considered elevated compared to samples of healthy controls.

### Immunohistochemistry

Human gingival samples were fixed in PFA 4% for 48 h, dehydrated in an increasing percentage of ethanol and embedded in paraffin. Immunohistochemistry was performed on 4-μm-thick sections. Antigens were retrieved by boiling slides in Tris-EDTA buffer. Primary antibody mouse anti-human IL-36γ (1:50, Proteintech, UK) was incubated overnight at 4 °C. Secondary antibody goat anti-mouse (1:200, Dako, UK) was used (30 min at room temperature). Specific binding was detected using 3,3-diaminobenzidine chromogen (Dako, UK). Sections were counterstained with Harris hematoxylin and mounted in Eukitt®. Automated whole-slide imaging was performed using the NanoZoomer 2.0 (Hamamatsu, Japan). The negative control was done by omitting primary antibody.

### Western blotting

Cells were washed twice with ice-cold PBS and then lysed in ice-cold lysis buffer (RIPA buffer: 50 mM Tris-HCl pH 7.5, 150 mM NaCl, 0.5% sodium deoxycholate 10%, 1% NP40, 0.1% SDS 20%*)* containing protease inhibitor cocktail (1:100, Sigma-Aldrich) on ice for 60 min. The lysate was centrifuged at 12,000 × *g* for 10 min at 4 °C and the supernatant containing the protein extracts was collected and stored at −80 °C until use.

For the protein isolation from cell culture media, supernatants were concentrated 25-fold using the Centricon® centrifugal filter (Millipore, USA). The protein concentration in the cell lysates and in the concentrated cell culture media was assessed using the Pierce® BCA Protein Assay Kit (Thermo scientific, USA). Protein extracts were diluted with Laemmli loading buffer containing β-mercaptoethanol before SDS-PAGE. Proteins were transferred to PVDF membrane and blocking was performed in blocking buffer (5% nonfat dry milk in TBST) for 1 h at room temperature. Primary antibody rat anti-human IL-36γ (MAB 2320, 1:2,000, R&D, USA) was incubated in blocking buffer overnight at 4 °C. Then secondary horseradish peroxidase conjugate antibody donkey anti-rat (712-035-153, 1:10,000, Jackson Immuno Research, UK) was incubated for 1 h at room temperature. The signal was detected using SuperSignal® West Dura Extended Duration Substrate (Thermo Fisher Scientific, USA) and the ChemiDoc Imaging SystemTM (Bio-Rad, USA).

### Statistical analysis

Results are given as means ± s.e.m. GraphPad Prism 5.0 software (GraphPad Software, USA) was used to perform nonparametric tests to compare data (the Kruskal-Wallis test followed, if significant, by group comparisons with the Mann-Whitney test) or for the Spearman correlation test. The n for statistical comparisons is the number of periodontitis patients or healthy controls includes in the *in vivo* studies as well as the number of healthy controls to extract primary cells and thus the number of *in vitro* biological replicates. The results were considered statistically significant if the *p*-value was less than 0.05. All experiments were repeated at least three times.

## Supplementary information

Supplementary informations

## Data Availability

The datasets used and/or analyzed during the current study are available from the corresponding author on reasonable request.
