# Supplementary material for: IL-36γ is a pivotal inflammatory player in periodontitis-associated bone loss
Source: Sci Rep. 2019 Dec 17;9:19257. doi: 10.1038/s41598-019-55595-9 (PMC6917751; doi:10.1038/s41598-019-55595-9)
Supplement: Supplementary file 1 — Supplementary informations [file 41598_2019_55595_MOESM1_ESM.docx]

**Supplementary informations**

IL-36γ is a pivotal inflammatory player in periodontitis-associated bone loss

Alexandra Cloitre^1,2,3^, Boris Halgand^1,2,3^, Sophie Sourice^1,2^, Jocelyne Caillon^4^, Olivier Huck^5,6,7^, Isaac Maximiliano Bugueno^5,6^, Fareeha Batool^5,6^, Jérôme Guicheux^1,2,3*^, Valérie Geoffroy^1,2^‡ and Philippe Lesclous^1,2,3^‡^*^

1 Inserm, UMR 1229, RMeS, Regenerative Medicine and Skeleton, Université de Nantes, ONIRIS, Nantes, France

2 Université de Nantes, UFR Odontologie, Nantes, France

3 CHU Nantes, PHU4 OTONN, Nantes, France

4 EA 3826 Thérapeutiques cliniques et expérimentales des infections, Nantes, France

5 INSERM (French National Institute of Health and Medical Research), UMR 1260, Regenerative Nanomedicine (RNM), FMTS, Strasbourg, France

6 Université de Strasbourg, Faculté de Chirurgie-dentaire, Strasbourg, France

7 Hôpitaux Universitaires de Strasbourg, Pôle de médecine et chirurgie bucco-dentaire, Department of Periodontology, Strasbourg, France

‡These authors are co-last authors on this work

^*^Correspondance : Jérôme Guicheux : [jerome.guicheux@inserm.fr](mailto:jerome.guicheux@inserm.fr)

**Supplementary Table S1. Patient characteristics.** NA: not applicable, Variables are shown as mean values ± s.e.m.

| **Variables** | **Healthy controls** | | **Periodontitis patients** | |
| --- | --- | --- | --- | --- |
|  | **Number** | **%** | **Number** | **%** |
| **Gender** |  |  |  |  |
| Male | 7 | 43.8 | 12 | 60 |
| Female | 9 | 56.3 | 8 | 40 |
| **Age (years)** | 21.1 ± 3.8 | | 50.5 ± 9.9 | |
| **Tobacco** |  |  |  |  |
| User | 4 | 25 | 12 | 60 |
| Non-user | 12 | 75 | 8 | 40 |
| **Probing pocket depth (mm)** | NA | | 5.4 ± 1.7 | |
| **Clinical attachment loss (mm)** | NA | | 6.8 ± 1.8 | |
| **Bleeding on probing** | 0 | 0 | 20 | 100 |

**
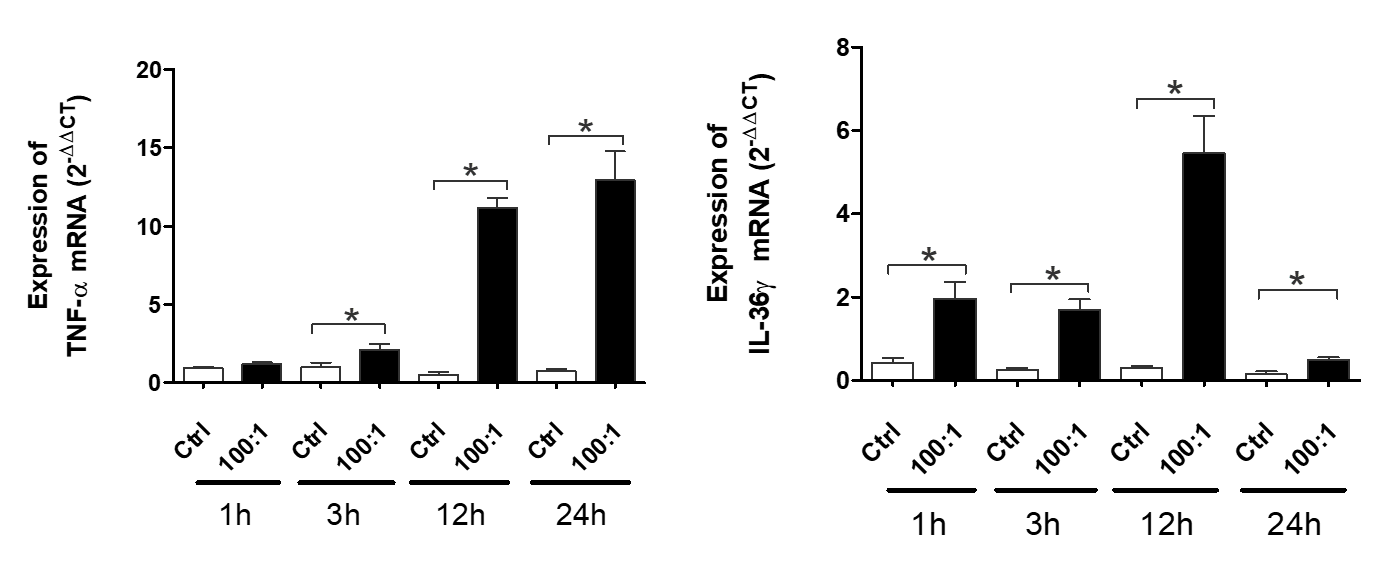
**

**Supplementary Figure S1. Effect of *Porphyromonas gingivalis* (*Pg*) infection on *IL-36γ* mRNA expression in human OKF6/TERT2 cells**. Human OKF6/TERT2 cells were cultured without *Pg* (control; Ctrl) or with *Pg* at 100:1 MOI for 1, 3, 12 or 24 h. TNF-α (positive control) and *IL-36γ* mRNA expressions were measured by RT-qPCR. Data are shown as mean ± s.e.m. 3 biological replicates of OKF6/TERT2 cells were used. n was used for statistical comparisons; **p*<0.05.

**
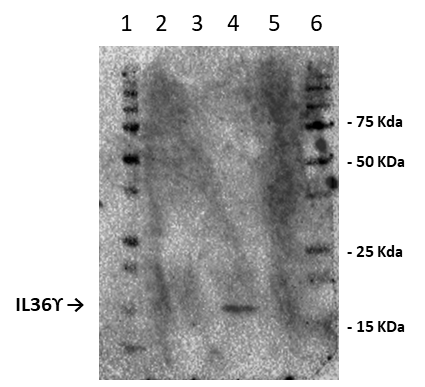
**

**Supplementary Figure S2. IL-36γ protein expression was assessed** **by Western blotting in the supernatants of human primary OEC culture**. 1- Ladder, 3- Ctrl 24 h without *Pg* stimulation, 4-stimulated OEC by *Pg* + (100:1 MOI 24 h), 6- Ladder. Lanes 2 and 5 are empty. Supernatants were subjected to Western blotting with anti–IL-36γ. The Western blot presented is representative of two independent experiments

**Supplementary Table S2. Variation of expression of the IL-36 cytokines in patients with psoriasis, rheumatoid arthritis (RA), Crohn’s disease (CD) and periodontitis compared to their respective controls and percentages of diseased patients with an elevated alternative *IL-36* agonists/antagonists ratio (*IL-36Ra* and *IL-38*) (>1.5).** Control for psoriasis was healthy skin, control for RA was synovial tissue of patients with OA, control for CD was colonic biopsies of patients with CD from normal uninflamed area, and control for periodontitis was healthy gingiva. For each cytokine, data are shown as expression variation (↗increase, ↘decrease, = steady), *p*-value (*p*) and fold change (x).

| **Ref** | **Chronic inflammatory disease** | **Number of samples (Control, Disease)** | ***IL-36α*** | | | ***IL-36β*** | | | ***IL-36γ*** | | | ***IL-36Ra*** | | | ***IL-38*** | | | ***Alternative IL-36* agonists/**  **antagonists ratio** **(*IL-36Ra* and *IL-38*) >1.5** |
| --- | --- | --- | --- | --- | --- | --- | --- | --- | --- | --- | --- | --- | --- | --- | --- | --- | --- | --- |
| Boutet et al. 2015 | Psoriasis (Pso) | 29 (14, 15) | **↗** | *p*=0.002 | x72 | **=** | ns | _ | **↗** | *p*=0.0001 | x11 | **↗** | *p*=0.0002 | x2.8 | **↘** | *p*=0.002 | x0.4 | 93% |
|  | Rheumatoid arthritis (RA) | 23 (6, 17) | **↗** | *p*=0.03 | x4.6 | **↗** | *p*=0.015 | x390 | **↗** | *p*=0.001 | x48 | **=** | ns | _ | **↗** | *p*=0.02 | x7.4 | 17-29% |
|  | Crohn's disease (CD) | 31 (15, 16) | **↗** | *p*=0.002 | x2.7 | = | ns | _ | **↗** | *p*=0.08 | x2.2 | **=** | ns | _ | **↗** | *p*=0.03 | x1.6 | 25% |
| This study | Periodontitis | 36 (16, 20) | **=** | ns | _ | **↘** | *p*=0.0013 | x0.4 | **↗** | 0.0027 | x2.9 | **↘** | *p*=0.0032 |  | **=** | ns | x0.4 | 65% |

**Supplementary Table S3. Primer sequences used for qPCR.**

| **Human genes** | **Forward (F) and reverse (R) primers** |
| --- | --- |
| *SDHA* | F: 5'-TGGGACAAGAGGGCATCTG-3'  R: 5'-CCACCACTGCATCAAATTCATG-3' |
| *B2M* | F: 5’-TTCTGGCCTGGAGGCTATC-3’  R: 5'-TCAGGAAATTTGACTTTCCATT-3' |
| *KRT14* | F: 5’-GTCAATGTGGAGATGGACGC-3’  R: 5'-GTGAAGAACCATTCCTCGGC-3' |
| *CD90* | F: 5’-CGAACCAACTTCACCAGCAA-3’  R: 5'-TCTGAGCACTGTGACGTTCT-3' |
| *IL-1β* | F: 5’-CCGGGACTCACAGCAAAA-3’  R: 5'-GGACATGGAGAACACCACTTG-3' |
| *IL-6* | F: 5’-TCCACAAGCGCCTTCGGTCCAG-3’  R: 5'-CTCAGGGCTGAGATGCCGTCG-3' |
| *TNFα* | F: 5’-CAGCCTCTTCTCCTTCCTGAT-3’  R: 5'-GCCAGAGGGCTGATTAGAGA-3' |
| *IL-17A* | F: 5’-AAGGCCCCTCAGAGATCAAC-3’  R: 5’-CCTTTCTGGGTTGTGTGGTG-3’ |
| *RANKL* | F: 5’-GCCAGTGGGAGATGTTAG-3’  R: 5'-TTAGCTGCAAGTTTTCC-3' |
| *OPG* | F: 5’-GGCAACACAGCTCACAAGAA-3’  R: 5'-CGCTGTTTTCACAGAGGTCA-3' |
| *IL-36α* | F: 5’-GAAAATTGACACACCTCAGC-3’  R: 5'-AGAGATTGAGTCCATTCAGG-3' |
| *IL-36β* | F: 5’-TGTGGGTCCTGAGTGGAAAT-3’  R: 5'-TCTGTGTCTCTACAGGCTATTAAATGA-3' |
| *IL-36γ* | F: 5’-CATTGCCTCCTCCAAGAGAG-3’  R: 5'-AGACCAAGCTGCCACCTCTA-3' |
| *IL-36Ra* | F: 5’-CATGGAGCTCTATCTTGGTG-3’  R: 5'-CTGGGTGAGTCTGACAGG-3' |
| *IL-38* | F: 5’-CCCCATGGCAAGATACTAC-3’  R: 5'-CCTCTTCTGTCTCCACACAT-3' |
| *TLR2* | F: 5’-GGTCATCATCAGCCTCTCCA-3’  R: 5'-AAAGATCCTGAGCTGCCCTT-3' |
| *TLR4* | F: 5’-GCAATGCTCCTTGACCACAT-3’  R: 5'-CTGTTTCTGAGGAGGCTGGA-3' |
| *IL-36R* | F: 5’-GCTGGAGTGTCCACAGCATA-3’  R: 5'-GCGATAAGCCCTCCTATCAA-3' |
